# Early Outcome Analysis of Management of Closed Pelvic Ring Fractures in Emergency: Conservative Versus Surgical at Level III Trauma Center in India

**DOI:** 10.7759/cureus.26195

**Published:** 2022-06-22

**Authors:** Sanket Mishra, Deepankar Satapathy, Nego Zion, Udeepto Lodh

**Affiliations:** 1 Department of Orthopaedic Surgery, Institute of Medical Sciences (IMS) and SUM Hospital, Bhubaneswar, IND

**Keywords:** closed pelvic fracture, external fixator, d’aubigne-postel, shock, pelvis

## Abstract

Introduction: Pelvic bone fracture is often observed in high-speed road traffic accidents, and forms a medical emergency as it is often complicated with associated internal exsanguination, shock, and mortality. Managing such cases cost-effectively in a developing country with limited assets, without compromising on patient outcomes still remains an obstacle.

Objective: To compare and contrast the clinical aftermath of urgent non-surgical and surgical treatment of closed pelvic ring fracture patients and to analyze the types and severity of complications and final functional outcome.

Material and methods: Twenty-five patients with pelvic fractures received at the casualty of IMS and SUM Hospital, Bhubaneswar, between January 2017 and January 2018 were included in the study. Marvin Tile classification was used to classify the fractures. Analysis and assessment of patients were done preoperatively and at six-month follow-up after management, with radiology and functional score using D’Aubinge-Postel Scale. The mode of injury, various management protocols for each type of fracture pattern, and associated complications were also noted. And finally, an outcome comparison was drawn between surgical and non-surgical options for various fractures. A Chi-square test was used to compare the outcomes.

Results: The functional outcome as per the D'Aubigne-Postel Scale, on average six months, was excellent in nine patients (36%), good in seven (28%), fair in four (16%), and poor in four (16%). The outcome comparison was insignificant statistically in both radiological assessment (p 0.614) and functional scores (p 0.26) between the conservative and surgical outcomes. The average duration of hospital stay, duration to ambulation, duration to full recovery, and complications were significantly more in patients managed conservatively. While the cost of treatment was more in the surgical group. One death was observed in the study group due to septicemia which might have been directly related to the severity of pelvic injury and choice of treatment.

Conclusion: Tile’s Type B and C fractures, managed surgically allow faster mobilization of the patient and a shorter recovery period while the cost of treatment is significantly more. Tiles type A is best managed conservatively.

## Introduction

The pelvic ring connects the upper half and lower half of our body and helps in distributing and transmitting the weight from the spine to the lower extremities. in addition with its unique shape, it houses and protects vital internal organs of the pelvis region and its flat external surface serves as an attachment for multiple muscles which keep sustaining weight and stability of our body [1]. The pelvic ring is formed by the connection of the sacrum to innominate bone at sacroiliac joints and the symphysis pubis. The bony pelvis is divided into True and False pelvis with the line of demarcation as the pelvic brim. The true pelvis contains the pelvic organs- bladder, urethra, rectum, uterus and vagina in females, and prostate in males. The false pelvis forms the lower part of the abdominal cavity. the two half pelvic bones called OS innominatum are fixed with each other to form the two sacroiliac joints posteriorly. the vertical orientation of both these joints subjects them to constant sheering force during various body activities primarily due to the weight of the body. such sheer is primarily resisted by the strong ligament complex comprising sacroiliac, sacrospinous and sacrotuberous ligaments. The pelvic brim is a crest of compact cancellous bone which is an approximately apple-shaped line passing through the promontory of the sacrum, the arcuate line and illio pectineal lines, and the superior brim of the pubic symphysis. The integrity of the pelvic brim is of paramount importance for pelvic ring biomechanics. All loads use this route and thus it is an important part of the management of pelvic trauma to attempt to repair and reconstruct it to its near anatomical shape in case of traumatic pelvic injury. Any fracture that alters the normal anatomical integrity and stability of this bony ring is considered an unstable pelvic fracture [2]. Traumatic forces, depending on their direction and strength, cause varied patterns of fracture ranging from simple two-part fractures to complex comminuted fractures with significant displacement or avulsion fractures, with or without superior-inferior transposition, accompanied or unaccompanied with dislocation of the pelvic ring [3,4].

Traumatic pelvic injuries account for 2% of all orthopedic hospital admission and nearly 3% of all skeletal fractures. a bimodal distribution in the age group of patients with such injuries is seen with one group comprising 20-40 years and the other group being elderly individuals older than 65 years. Patients of polytrauma can have pelvic injuries ranging from 3% to 82% out of which 13% to 17% are inherently unstable pelvic ring fractures. Most pelvic fractures in young occur with a high-energy mechanism of injury. Therefore, they are often combined with other injuries [5]. The most common high-energy injury is motor vehicle accidents. Patients who sustain these injuries not only have the osseous injury but also often have concomitant life-threatening injuries including blunt trauma abdomen, hollow viscus perforation, genitourinary injuries, chest injuries, spinal injuries, and head injuries. Early death after these injuries is usually due to hemorrhage, multiple organ system failures, or sepsis (as high as 40% to 50%) [6].

Simple and fast radiographs with a normal anterior-posterior view of the pelvis with both hips or special inlet and outlet pelvic views, provided a very good insight into the pattern of pelvic fractures most times. the more elaborate Judet pelvic views were not done considering the difficulty in patient positioning and time factor. simple radiography can diagnose efficiently above ninety percent of all pelvic fractures [7]. Computed tomography (CT) imparts better information on the patterns of pelvic injury especially in doubtful cases, while magnetic resonance (MRI) is done to diagnose accompanied pelvic organ injury including deep vein thrombosis [8].

Once conferred with a case of pelvic injury the first job of the clinician is to save the life of the patient and stabilize his or her general condition. Pelvic bone fractures with associated injuries result in 30%-58% of mortality [9,10]. Pelvic fracture is the third most common cause of death in trauma [11]. Delayed recognition and inappropriate management of the trauma patient with a pelvic injury can lead to a poor and fatal outcome [2]. The ultimate aim of urgent treatment of pelvic ring injury is to reduce exsanguination, and management of associated injuries. Processes of achieving these aims have changed substantially. After all these years of research on such serious orthopedic injuries requiring urgent surgical stabilization, dissension still remains with respect to standard treatment protocols and outcomes [12] and the lack of availability of appropriate instruments and operative skills also adds to the constraint factor. In recent times, the focus is being shifted towards external fixation as it accomplishes the aims of emergency pelvic fracture management with less expertise, lesser time, and is minimally invasive yet provides pelvic stability and reduces exsanguination and soft tissue injury [5]. This might reduce the complications associated with pelvic fracture. This is of paramount importance in a level 3 trauma center in a country like India where the availability of specialized hospitals equipped to deal with complex pelvic surgical procedures is limited. If proper stabilization can be achieved at a lower-level hospital, it prevents economic loss to the patient and serves to save the life of the patient as well by preventing valuable wastage of time on transportation at the same time giving good functional outcomes.

## Materials and methods

Twenty-five patients with a pelvic injury who presented to the casualty of IMS and SUM Hospital, a level III trauma center in Eastern India, between January 2017 and January 2018 were included in the study. After the primary survey and stabilization, a secondary survey was carried out and radiographic assessments were done. Marvin Tiles classification was used to classify the fracture. Patient selection for the study was based on predetermined inclusion and exclusion criteria.

After the primary and secondary survey and stabilization of a general condition, definite management of the patients was planned. Sixteen patients were offered conservative treatment (tile A and Tile B1) which included pelvic binder application, bed rest, bedsore care, bladder bowel care, etc. Six cases of Tile B were managed with stabilization with an external fixator with or without postoperative traction. Two cases of tile B refused surgery and were managed conservatively. Three cases of Tile C were managed with internal fixation. Two cases of tile C refused surgery and refused transfer to a higher setup and were managed conservatively. All cases were followed up for an average of six months. Functional and radiological follow-up scoring was done using the D’Aubigne-Postel score and Slatis radiological grading. The scores were compared using a standard chi-square and a p-value of 0.05 was taken as significant.

Inclusion criteria

Case of closed pelvic injuries with or without polytrauma. All patients with closed pelvic injury who gave written consent to participate in the study. Pelvic fractures of all age groups and all causes.

Exclusion criteria

Open pelvic injuries, cases with severe multisystem injuries that had to be referred urgently to higher set up due to lack of infrastructure at our level 3 set up, patients who refused consent, patients with pathological fracture, and patients with associated acetabulum fracture.

Operative procedure

**Figure 1:**
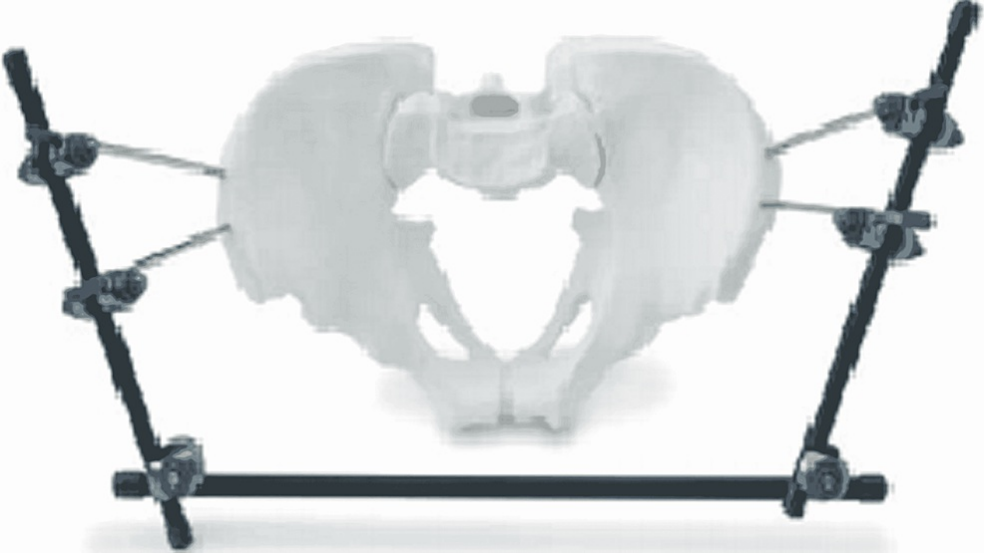
Iliac crest frame

**Figure 2:**
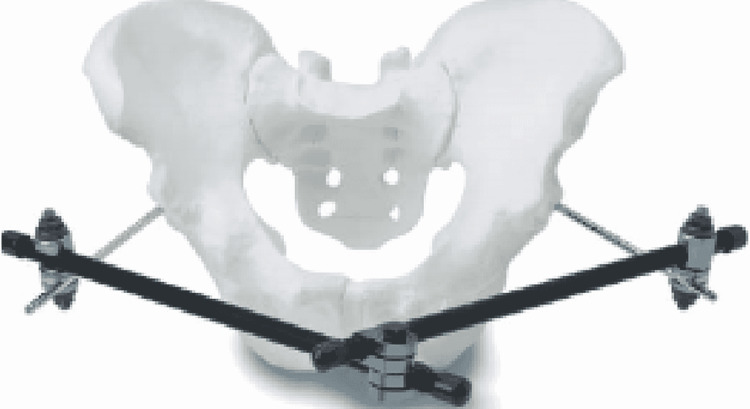
Supraacetabular pins

The surgical procedure consisted of the application of an external fixator which was used in cases of Tile’s Type B injuries, where there was superior-inferior translation and rotational instability. External fixation was also used in stable “open book” fractures, where radiological findings indicated the intact sacroiliac, iliolumbar, sacrospinous, and sacrotuberous ligaments. Two pins were put on the iliac crest or supraacetabular region and connected using following patterns of fixation as showed in Figures 1, 2.

**Figure 3:**
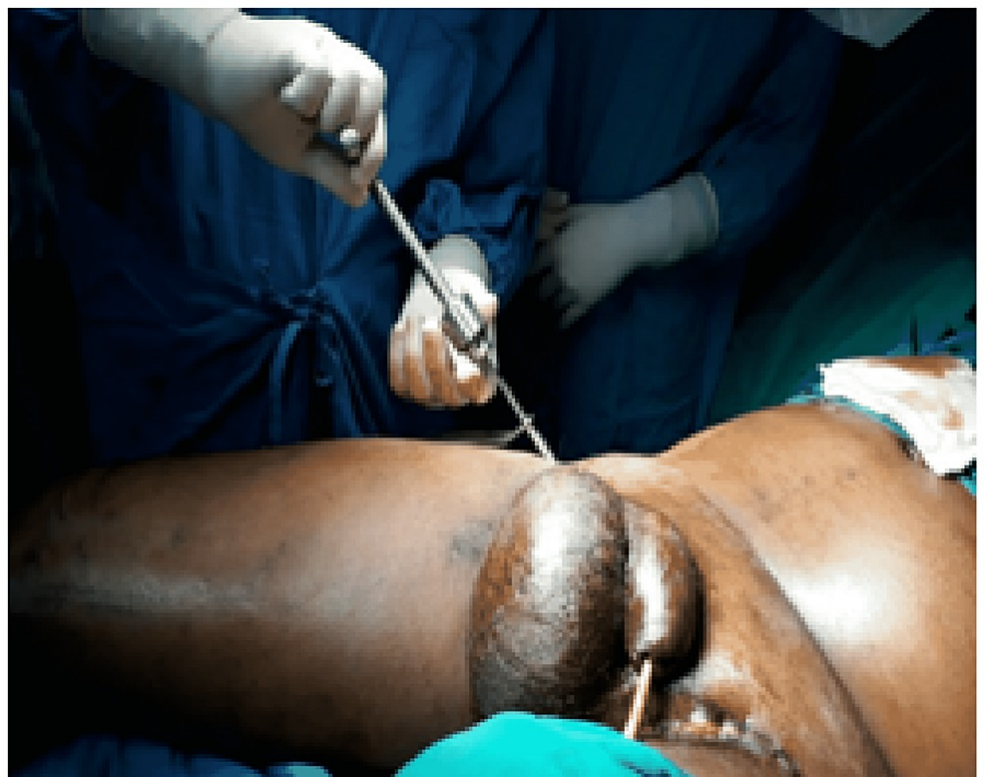
Fixation of iliac crest pins for pelvic external fixation system

**Figure 4:**
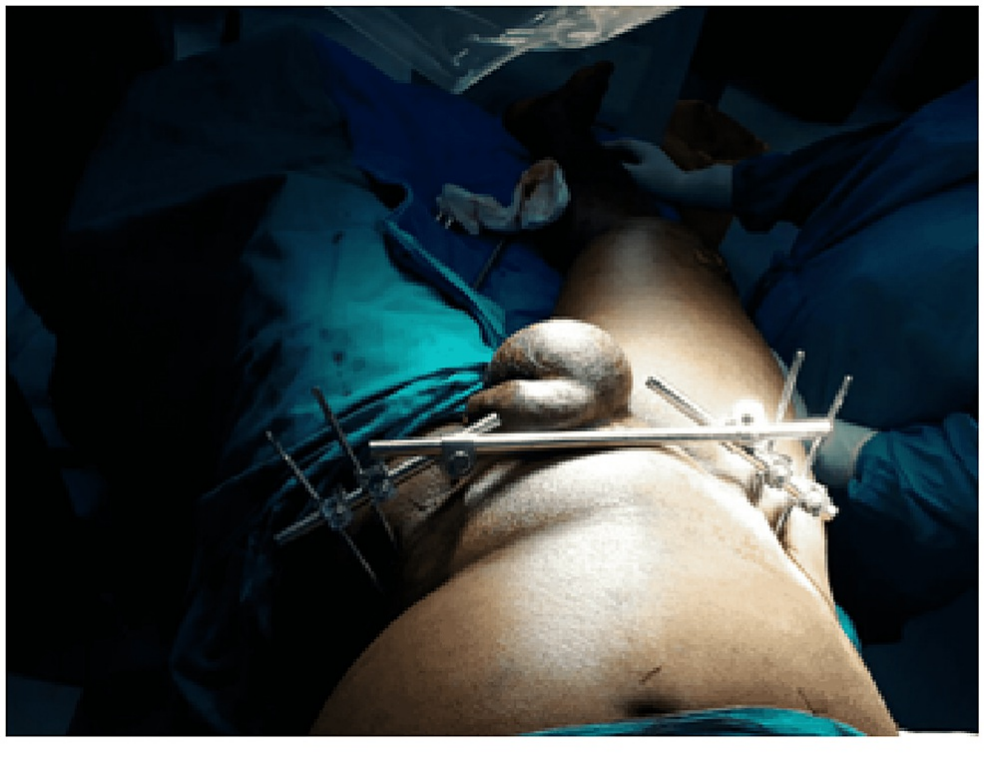
Fully fixed and stable pelvic external fixation system using iliac crest pins

Illustrative images of the external fixator application are shown in Figures 3, 4.

Open approaches depended on the type and location of the fracture. We used the Anterior Suprapubic approach for pubic diastasis, the Posterior approach to the sacroiliac joint, Anterior approach for exposing iliac wings. Standard AO spring (recon) plate with standard screw and calculated screws for sacrum were used for surgery. All cases were operated on under spinal anesthesia.

## Results

According to Marvin Tile’s classification, 12 patients (48%) suffered from Type A pelvic fractures, eight patients (32%) Type B, and five patients (20%) from Type C fractures. Our study showed that Type A fracture with a stable configuration of the pelvic ring formed the bulk of the patients (Table 1).

**Table 1:** Number of patients in each grade of TILE'S pelvic injury grade

TILE ‘S pelvic injury grade	No of patients	Percentage
A	12	48%
B	8	32%
C	5	20%
total	25	100%

Associated trauma

Out of our 25 cases, 18 cases (72%) were associated with polytrauma. Shock due to hypovolemia was seen in 16 (64%) cases, while associated head injury was present in 11 (44%) cases. Blunt trauma to the abdomen (36%) with urethral and lower pelvic visceral injuries accounting for 44% (11 cases) of associated injuries. Most patients of polytrauma had more than one system involvement (Table 2).

**Table 2:** Type of associated injury

Type of associated injury	No of cases	Percentage
Hypovolemic shock	16	64%
Head injury	11	44%
Spine injury	2	8%
Blunt trauma chest	3	12%
Blunt trauma abdomen	9	36%
Urethral & pelvic injuries	11	44%

Age group of injury: In our study 28% patients were between the age group of 20-35 years and 52% were between 35 years and 50 years. Mean age was 38.4 years (Table 3).

**Table 3:** Age group of injury

Age group	Number of cases	Percentage
<20 y	0	0%
20-35 y	7	28%
35-50y	13	52%
50-65 y	5	20%
>65 y	0	0%

Mode of injury

80% of our cases with 20 patients were caused due to road traffic accidents, while 12% were due to fall from height (Table 4).

**Table 4:** Mode of injury

Mode of injury	Number of cases	Percentage
Motor vehicle accident	20	80%
Fall from height	3	12%
Industrial injury	2	8%

Treatment received

All 12 cases of type A pattern of pelvis fracture received conservative treatment with rest, immobilization and medication. Medication included broad spectrum antibiotics, analgesics, anti-inflammatory drugs and DVT prophylaxis for prolonged immobilization. Out of eight patients of Tile type B, six underwent stabilization with an external fixator followed by similar medications. Out of five patients with Tile type C, three underwent Open reduction, while two refused surgery and were managed conservatively (Table 5).

**Table 5:** Treatment received

Fracture type	Conservative	Surgical Ext fixation ORIF	
Tile A 12	12	0	0
Tile B 8	2	6	0
Tile C 5	2	0	3
Total 25	16	6	3
percentage	64%	24%	12%

Radiographic outcomes

Six-month radiological follow-up of patients using Slatis scoring showed 60% patients with excellent outcomes and 16% with good scores (Table 6).

**Table 6:** Radiographic assessment as per SLATIS

Radiographic grading (slatis)	Conservative	Surgical	P-value
Excellent	12	3	0.6140
Good	2	2	
Fair	0	1	
Poor	1	3	
Death	1	0	
Total	16	9	

Functional outcomes

Functional scoring at six-month follow-up using D’Aubigne-Postel scoring showed overall 36% excellent results while 28% patients had good scoring. Poor results were seen only in 16% patients.

Outcome comparison

Comparison of conservative and surgical management at six-month follow-up based on radiological assessment of Slatis showed no statistically significant difference between the two methods (p 0.614). Similarly, the functional outcomes at six months were also similar with no statistical significance (p 0.2605).

Comparison between surgical and conservative managements: Average duration of hospitalization for patients managed conservatively was significantly higher with an average of 30 days compared to 15 days in the surgical group. Average duration needed for ambulation was significantly higher for conservative group with 60 days. Number of cases with complications was more in the conservative group. Duration for full recovery was significantly less than seven months in the surgical group compared to 12 months in the conservative group (Table 7).

**Table 7:** Comparison between surgical and conservative management

Parameters	Conservative	Surgical
No of patients	16	9
Avg duration of hospitalization (days)	30	15
Avg duration for sitting upright (days)	40	5
Avg duration for ambulation (days)	60	35
Pulmonary embolism	2	0
DVT	2	0
Average duration of full recovery (months)	12	7
Avg cost of treatment per person	17,000	37,000

## Discussion

Pelvic fractures are such rare injuries but still constitute 3%-8.2% of all traumatic injuries presenting in orthopedic emergencies [2]. and road traffic accident continues to remain the primary etiology, involving 70% of cases [13]. 13% to 17% of such pelvic fractures are inherently unstable [2]. The pelvic ring is a strong and stable framework with relatively high energy and forces such as those produced in road traffic and motor vehicle accidents are needed to disrupt the pelvic architecture and such high forces also cause concomitant other injuries including urogenital, abdominal spinal, and head injuries [5]. hence most of the time multidisciplinary and team work including neurosurgeons, anesthesiologists, orthopedic surgeons, and plastic surgeons, is needed with well-equipped and staffed setups to treat such patients. stabilization of vital parameters is the first priority with subsequent standardized steps to diagnose and treat respective fractures which include surgical fixation [14]. Suspected abdominal bleeding should be quickly evaluated with clinical examination and emergency ultrasound to identify major bleeding and hollow viscus injury (FAST) [15].

High mortality of 40%-50% is seen in pelvic fractures with hemodynamic instability. the morality is further higher with concomitant head injury [13]. exsanguination and intractable hemorrhage within the pelvis are believed to be a major cause of mortality while others believe such extensive hemorrhage is highly unlikely and associated injuries could result in higher mortality [12]. External fixation has been reported to be applied as early as 15 minutes by some orthopedic surgeons [10]. Such quick application helps in stabilizing the pelvic ring, thereby preventing shifting of fractured fragments, helps reduce relative pelvic volume, and decreases potential space for internal hemorrhage. If the patient continues to remain hemodynamically unstable, angiographic embolization can be considered or internal exploration might be needed [10]. This requires specialized operation theater and specialist surgeons and a team that may not be available at all times in developing countries like India.

Solomonet et al. in their work show that among 1,479 pelvic ring fractures, 1,029 cases had polytrauma with 10% having head injuries, 3% had spine injuries involving L5 nerve root and adjoining lumbosacral plexus. hemodynamic instability was encountered and addressed in 20% of cases [16]. There were 18 cases (72%) in our study that had polytrauma. Among them 11 (44%) suffered head injuries, three (12%) suffered blunt chest injuries, nine (36%) suffered blunt abdominal organ injuries and 11 (44%) patients had urogenital injuries. During admission, 16 patients (64%) had clinical and laboratory signs of hemorrhagic shock. A rapid ABG analysis with HB %, hematocrit, serum Ph estimation, lactate levels, electrolytes, serum creatinine, and BUN analysis helps to identify early metabolic decompensation and efforts to address them. Exsanguination and subsequent refractory shock were the leading cause of death in patients with pelvic ring disruption injuries [17]. 10%-15% of patients with severe pelvic injuries might have arterial injury leading to massive internal hemorrhage and some 7%-11% might need embolization to control the same [18]. Head injury including subdural and extradural hemorrhage form the second most common cause of death followed by abdominal injuries and delayed sepsis.

Management of pelvic injuries in a developing country with limited resources revolves around early patient stabilization and early patient selection for appropriate management. With limited resources and the economy, the right patients should be selected for referral to a higher setup. This prevents patient morbidity and reduces complications and costs. In more recent times, internal fixation with reconstruction plates and screws and external fixation have been used for the management and stabilization of unstable pelvic ring fractures. Kallan et al. used external fixation for all Type B pelvic ring fractures. With it, he achieved and maintained repositioning in 83% of pelvic fractures. Similarly, he used it successfully against 21% of Tile’s Type C1 fractures. Similarly, Tile et al. reported that among 494 pelvic fractures, unstable fractures (Type C) were seen in 92 patients (21%). Surgery using external fixators was done in 68 patients (13.76%) and with internal fixation in 24 patients (8.16%). The external fixator not only restores pelvic arch stability but also provides reduction and stability of the subluxated sacroiliac joint. Internal fixation for 24 patients with tile Type C was done with screws and plates. This stabilization method resulted in anatomic repositioning and early ambulation and weight-bearing.

In our study, Type A pelvic injury was seen in 12 patients (48%), Type B in eight patients (32%), and Type C in five patients (20%). Sixteen patients (64%) were treated conservatively (all 12Type A and 2 patients with Type B1). Two patients with Type C fracture did not provide consent for the surgery so were treated conservatively while the rest six Type B patients (24%) were treated surgically and three remaining Type C patients (12%) were managed with internal plate and screw fixation.

 Briffa et al. [19] reported the outcome of 161 of 257 surgically fixed acetabular fractures. excellent results were seen in 75 patients (47%), good results in 41 patients (25%), fair results in 12 (7%), and poor results in 33 (20%) were obtained using the D’Aubigne-Postel score. Poor prognostic factors included increasing age, delay in surgery, quality of reduction, and some fracture patterns. Similarly, Deo et al. [20] reported that in 79 patients with pelvic fractures, 74 subjects (94%) had to follow up for a mean period of two years and six months. Fifty-five subjects (74%) had an excellent outcome, which in turn is correlated to prompt surgical management and anatomical reduction. Poorer results were related to delayed surgical management, failure to maintain reduction, and femoral head damage during injury. Whereas Miranda et al. describe out of 218 patients with pelvic injuries managed conservatively about 60% had residual pain and 30% had to change their line of work to adjust to such a situation [21]. Berner et al. [22] reported 16% functional and 17% radiological unsatisfactory outcomes in 42 patients treated conservatively after combined pelvic symphysis and sacroiliac disruption injuries. However, with internal fixation of similar injuries, satisfactory results were obtained with 21% function and 27% radiological outcomes. He reported patients who managed conservatively did better in the long run. 10mm or more of residual fracture fragment displacement was considered critical and one of the reasons for prolonged pain in such patients. Lindahl et al., in their work, show good results are obtained in type B1 injuries managed with external fixation at the same time external Hoffmann fixation fails to maintain a bone reduction in type C pelvic injuries in 35% of patients [23].

In our study, 12 patients with Type A fracture were all managed conservatively, while out of eight cases of Type B, six were managed with external fixators and two cases of Type B1 were managed conservatively. Out of five cases of Type C, two cases were managed with ORIF with plates and screws, and 1 case was managed with a combination of External fixator and ORIF. two cases denied surgery and were managed conservatively. Postoperative follow-up radiological and functional assessment based on Slatis radiological grading and D’Aubigne-Postel score, respectively, showed an overall excellent score in 60% cases (15 cases) radiological and 36% cases (nine cases) functionally. Good scores were seen in 16% and 24% of cases, respectively. Poor scores were seen in 16% of cases both radiologically and functionally. On comparing both radiological and functional outcomes, the scores were statistically insignificant among the cases managed conservatively and surgically with a p-value of 0.614 and 0.2605, respectively. Our study showed if the patient selection is correct, then there are similar outcomes in cases managed conservatively and surgically. The low functional score may be the result of poor outcomes in Type C fractures who refused surgery and were managed conservatively and due to non-maintenance of reduction of open-book fractures due to pin tract infection in two cases. Inappropriate pin application in one and noncompliance to rehabilitation in one patient.

Latenser and Gentilelloin in their work with 37 patients with unstable pelvic fractures managed conservatively and surgically found that hospital stay was reduced in operated patients by 37.8%. they concluded that early surgical stabilization of pelvic injuries reduced blood loss, and hospital stay and reduces long-term disability while providing high survival rates [12]. In our study average duration of hospitalization was significantly higher in the conservative group (30 days) compared to 15 days in the surgical group. Patients in the surgical group significantly had early ambulation at 35 days compared to 60 days in the conservative group. The total cost of treatment was slightly more in the surgical group probably due to the higher cost of implants in the local market. The rate of complications like DVT and pulmonary embolism was higher in the conservative group due to prolonged immobilization. One case of death was also seen in a case of Type C fracture who denied surgery and was managed conservatively showing it is a poor choice of treatment for unstable pelvic fractures. Apart from the usual complications, inadequate pelvic fracture treatment also results in late complications manifested by chronic pain, unequal limb length, difficulty in walking, and neurological symptoms like lower limb radiculopathy and heterotrophic ossification, which were seen in a few cases.

Limitations

Our study is limited by less number of cases. With larger study group, there might be minor change in the outcomes of the study.

## Conclusions

After analysis of the outcomes of cases in our study, we can safely conclude that conservative treatment is the preferred modality for pelvic type A fractures, while external fixation provides excellent results in type B and internal fixation with or without augmentation with external fixation is the ideal treatment for type C pelvic injury cases. Surgical treatment allows faster mobilization of the patient, and shortens the recovery period, but is associated with limited availability, and increased cost as compared to non-surgical treatment. In a developing country like India with limited health resources, finances, and infrastructure, proper conservative management with comparable outcomes to surgical management can be a boon for the needy and poor patients who form the bulk of the patient load in a lower-level trauma center and peripheral centers.
